# Retraction: LncRNA MALAT1 aggravates MPP-induced neuronal injury by regulating miR-212 in SH-SY5Y cells

**DOI:** 10.1039/d1ra90069b

**Published:** 2021-02-01

**Authors:** Laura Fisher

**Affiliations:** Royal Society of Chemistry Thomas Graham House, Science Park, Milton Road Cambridge CB4 0WF UK advances-rsc@rsc.org

## Abstract

Retraction of ‘LncRNA MALAT1 aggravates MPP-induced neuronal injury by regulating miR-212 in SH-SY5Y cells’ by Dahua Yuan *et al.*, *RSC Adv.*, 2019, **9**, 690–698, DOI: 10.1039/C8RA09260E.

The Royal Society of Chemistry hereby wholly retracts this *RSC Advances* article due to concerns with the reliability of the data. The paper was analysed by experts who fact-checked the identities of the described nucleotide sequence reagents,^1^ and found errors with the following nucleotide sequence reagents reported in the article: miR-212 forward and reverse primers. The miR-212 primers would not be expected to target and amplify miR-212, and therefore the results in Fig. 4 are unreliable.

Given the significance of the concerns about the validity of the data, the findings presented in this paper are not reliable.

The authors state that there was a partial lack of rigour in the study and agree to retract the article in order not to mislead readers.

Signed: Laura Fisher, Executive Editor, *RSC Advances*.

Date: 19^th^ January 2021.

## Supplementary Material
